# Cat’s whiskers (*Orthosiphon stamineus*) tea modulates arthritis pathogenesis via the angiogenesis and inflammatory cascade

**DOI:** 10.1186/s12906-016-1467-4

**Published:** 2016-11-24

**Authors:** Yasser M. Tabana, Fouad Saleih R. Al-Suede, Mohamed B. Khadeer Ahamed, Saad S. Dahham, Loiy E. Ahmed Hassan, Saba Khalilpour, Mohamad Taleb-Agha, Doblin Sandai, Aman S. Abdul Majid, Amin Malik Shah Abdul Majid

**Affiliations:** 1 Faculty of pharmacy, University Technology Mara (UiTM), Bertam campus, Penang, Malaysia; 2 EMAN Research and Testing Laboratory, School of Pharmaceutical Sciences, Universiti Sains Malaysia, Minden, 11800 Pulau Pinang Malaysia; 3 Department of Pharmacological and Biomolecular Sciences, University of Milan, Milan, Italy; 4 Advanced Medical and Dental Institute (IPPT), Universiti Sains Malaysia, Bertam, 13200 Kepala Batas, Penang Malaysia; 5 Department of Pharmacology, Quest International University, Perak, Malaysia

**Keywords:** Anti-arthritis, Anti- inflammatory, *Orthosiphon stamineus*, Fluorescence Molecular Tomography (FMT), Pro-inflammatory mediators

## Abstract

**Background:**

*Orthosiphon stamineus* is used traditionally to treat gout, arthritis, and inflammatory related conditions. The in vitro anti-inflammatory effects of the plant have been scientifically investigated. The goal of the present study was to evaluate the potential of the 50% ethanol extract of *O. stamineus* (EOS) to treat rheumatoid arthritis.

**Methods:**

Anti-arthritic activity was assessed using the in vitro heat denaturation test and the (FCA)-induced arthritis model. Efficacy was assessed by measurements of paw edema and granulation, X-ray radiography, fluorescence molecular tomography (FMT), and histological evaluation. Levels of (TNF-α), interleukin-1 (IL-1), and (COX-1 and COX-2) were analyzed in vitro in lipopolysaccharide (LPS)-stimulated human macrophage (U937). TNF-α and IL-1 levels in the serum samples of arthritic rats were also measured using an ELISA kit.

**Results:**

Treatment with EOS resulted in dose-dependent inhibition of paw edema in acute and chronic models of inflammation. It also inhibited significantly the production of TNF-α, IL-1 COX-1, and COX-2 in the LPS-stimulated U937 macrophages. EOS significantly suppressed FCA-induced paw edema as well as the serum levels of TNF-α and IL-1. X-rays of the synovial joint of the hind leg showed considerable improvement in joint integrity and recovery of tibia-talus bones from degeneration and osteoporotic lesions. Histology of proximal interphalangeal joints of EOS-treated animals showed obvious protection of cartilage and soft tissue. Finally, FMT analysis strongly supported the anti-arthritic effect of EOS. EOS had high phenolic and total flavonoid content as well as strong antioxidant activity.

**Conclusions:**

Results illustrated that the anti-arthritic properties of *O. stamineus* could be beneficial for prevention and management of rheumatoid arthritis and other chronic inflammatory disorders.

**Graphical abstract:**

Illustration of the Anti- arthritis efficacy of Orthosiphon Stamineus standardized extract. 
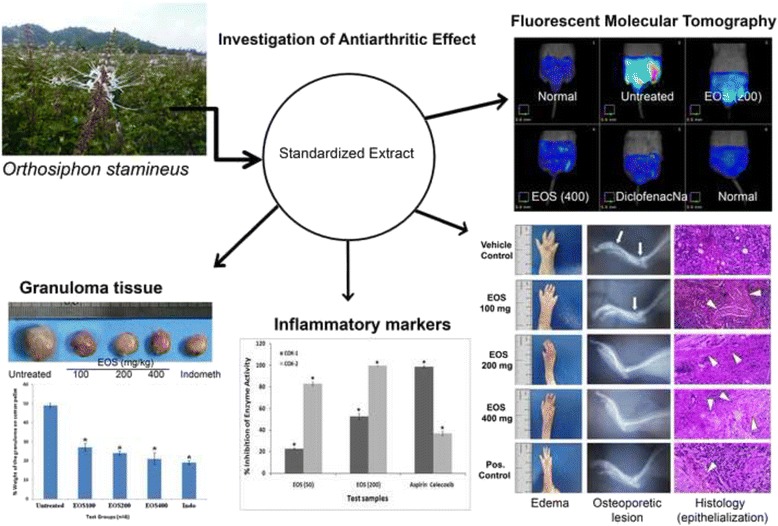

## Background

Arthritis is an autoimmune disorder that encompasses a group of systemic diseases that target and attack the articular joints of the body, leading to erosive inflammation in the synovial membranes. Arthritis is considered to be one of the leading causes of disability in people of all ages [1, 2]. Rheumatoid arthritis (RA) is a consequence of erosive synovitis, neovascularisation, and synoviocyte hyperplasia, which lead to articular cartilage and joint destruction [3]. Recently, the number of people diagnosed with arthritis has significantly increased to 60 million cases worldwide, with more women being affected than men, and 60–90% of patients use herbal remedies to treat their symptoms. The use of traditional medicinal herbs and alternative medicines is on the rise because of the many severe side effects associated with use of non-steroidal anti-inflammatory drugs (NSAIDs), including the risk of gastrointestinal and cardiovascular complications [4]. Standardized herbal extracts work on multiple targets to both cure the disease and avoid the side effects of NSAIDs.

*Orthosiphon stamineus* Benth. (Lamiaceae), locally known as “Misai Kucing” or cat’s whiskers, is a traditional medicinal herb used in southeast Asia to treat various inflammatory diseases such as cancer, hepatitis, rheumatism, abdominal pain, psoriasis, hyperlipidemia, diabetes, and kidney stones [5]. In Europe, people use a decoction of leaves of *O. stamineus* to make java tea to improve general health and fitness [6]. Scientific studies have reported the presence of bioactive pentacyclic triterpenes, betulinic acid, oleanolic acid, ursolic acid, β-sitosterol, and more than 20 other phenolic compounds in the leaves of this plant [7]. Recently, the potent antiangiogenic activity of *O. stamineus* and its prevention activity against human breast tumors in a xenograft model were reported [8]. Another study reported that the ethanol extract of *O. stamineus* specifically inhibited vascular endothelial growth factor (VEGF) expression and VEGF receptor (VEGFR) phosphorylation, which are known to be up-regulated during new blood vessel formation. *O. stamineus* also suppressed vascularization and inhibited growth of implanted human colon tumors. The high amount of rosmarinic acid in the ethanolic extract of *O. stamineus* played a main role in these activities [9]. The ant-inflammatory properties of *O. stamineus* leaf chloroform extract have been scientifically proven, it shows a potent in vitro inhibition to pro-inflammatory mediators expression such as iNOS, COX-2 and TNF-α, as well as PGE2 and NO production [10]. In addition, flavonoid rich chloroform extract fraction of *O. stamineus* inhibits the production of prostaglandin and NO, the activity ascribed to the rich contents of flavonoids, sinensetin and eupatorin,. Treatment with the fraction causes reduction of Rat hind paw edema [11]. In vivo treatment with *O. stamineus* demonstrated significant inhibitions activities against TPA -induced inflammation to mouse ears [12]. Another in vivo study using hexane fraction of *O. stamineus* confirmed the potent inhibition of carrageenan induced paw edema in rat [13, 14]. To the best of our knowledge, there is no anti-inflammatory, in vitro and in vivo anti-arthritis have been reported using 50% ethanol extract.

The objective of this study was to investigate the anti-arthritis, antioxidant, and anti-inflammatory properties of the 50% ethanol extract of *O. stamineus* leaves (EOS). The mechanisms that underlie the anti-arthritic properties of EOS were assessed using in vitro and in vivo animal models, and fluorescence molecular tomography (FMT) and X-ray in vivo imaging were used to evalute the anti-arthritic efficacy of the extract. The carrageenan-induced rat paw edema model for acute inflammation and the cotton pellet-induced granuloma model for sub-chronic inflammation were used to evaluate the effects of EOS on inflammation. Total flavonoid,Total phenolic, DPPH, ABTS and FRAP used to determine the antioxidant efficacy of EOS.

## Methods

### Cell culture reagents and conditions

The U937 human macrophage cell line and fetal bovine serum (FBS) were purchased from the American Type Culture Collection (Masassas, VA, USA). RPMI 1640 and penicillin/streptomycin were procured from Invitrogen (Carlsbad, CA, USA). Potassium chloride (KCl) and hydrochloric acid (HCl) were obtained from BDH Chemicals Ltd. (Poole, United Kingdom). PMA (phorbol 12-myristate 13-acetate), 2,2’-diphenyl-1-picrylhydrazyl (DPPH), gallic acid, Folin-Ciocalteu reagent, sodium carbonate (Na_2_CO_3_), lipopolysaccharide,and quercetin were purchased from Sigma Aldrich (Germany). Aluminum chloride (AlCl_3_), potassium acetate, sodium nitrate, sodium hydroxide, and 2,2-azinobis (3-ethylbenzothiazoline-6- sulfonic acid) (ABTS) were purchased from Merck (Germany). Diammonium salt, 2,4,6-Tri (2- pyridyl)-s-triazine (TPTZ), ferric chloride (FeCl_3_), and potassium persulfate were obtained from Sigma Aldrich (Poole, Dorset, UK). Chloroform was obtained from R & M Marketing (Essex, UK). Methanol and absolute alcohol were purchased from Riedel-de Haën (Seelze, Germany), and λ-carrageenan was procured from Sigma Aldrich (St. Gallen, Switzerland).

### Experimental animals

Male Sprague Dawley rats (150–200 g) were obtained from the Animal Research and Service Centre, Universiti Sains Malaysia (USM). The animals were kept in the animal transit room at the School of Pharmaceutical Sciences, USM (12 h dark/light cycle, 40–60% relative humidity, and 25 °C temperature). The animals were provided free access to food and water. However, the food was withdrawn 12 h before any experimental procedure was conducted on the animals. The experimental work was consistent with guidelines of the USM Committee for Animal Care and received approval from the USM Animal Ethical Committee (USM/PPSF/50 (084) Jld.2).

### Plant material and extraction procedure

Leaves of *O. stamineus* were obtained from a contract farming facility in Penang, Malaysia. *O. stamineus* were studied and confirmed by the Senior Botanist Mr. Shanmugan, School of Biological Sciences, USM. The specimen (Voucher No.: 11009) was deposited at the herbarium of the School of Biology, USM. The 50% ethanol extract of the O. stamineus leaves (EOS) was prepared using the maceration process as previously described [8].

### Phytochemical study of EOS

#### Determination of total phenolic content

Folin-Ciocalteu reagent was used to determine the total soluble phenolic content of EOS. Gallic acid was used as the standard according to the method of [15].

#### Determination of total flavonoid content

The AlCl_3_ colorimetric method was used to determine the total flavonoid content of EOS, and quercetin was used as the positive control as described before [16].

#### Ferric reducing antioxidant power (FRAP) assay

The antioxidant capacity of EOS was determined using the FRAP assay following the method described by [17].

#### ABTS assay

The ABTS radical scavenging capacity assay was conducted according to the procedures described by [18, 19].

#### Free radical scavenging (FRS) activity

The antioxidant activity of EOS also was evaluated by measuring its ability to scavenge the stable free radical DPPH following a previously described technique with minor modifications [20].

### In vitro anti-inflammatory assay of effects of EOS

#### Cell proliferation assay

The MTT assay was used to assess the effect of EOS on U937 cells using a previously described technique with minor modifications [21–23].

#### Cell viability assay

Viability of cells was determined using the trypan blue exclusion method as described before [24, 25].

#### Analysis of cytokine levels in differentiated human macrophages

Differentiated human macrophages (100 μl, 2 × 10^5^ cells/ml) were seeded into 96-well flat-bottom plates in the presence or absence of LPS (1 μg/mL) for 24 h. The macrophages were treated with 100 μl of various concentrations of EOS (12.5, 25, 50, 100, and 200 μg/ml). The plates were incubated at 37 °C under 5% CO_2_. After 48 h, the plates were centrifuged at 1000 rpm for 10 min. Next, 100 μL of supernatant were used to measure the concentration of interleukin 1 (IL-1) and tumor necrosis factor (TNF-α). The optical densities of the samples were measured using the Tecan Infinite pro200 microplate reader at 550 nm. The calibration curves of standards were used to calculate the concentration of IL-1 and TNF-α in the samples [25].

#### In vitro cyclooxygenase (COX) inhibition assay

The effect of EOS on COX was assessed using enzyme-based kits purchased from Cayman Chemicals (Ann Arbor, MI, USA) according to the method described before [26].

#### In vivo acute anti-inflammatory assay

The acute anti-inflammatory effect of EOS was evaluated using the carrageenan-induced rat hind paw edema model as mentioned before [27].

#### In vivo sub-chronic cotton pellet granuloma model in rats

Granuloma tissue formation was used to study the anti-inflammatory effect of EOS following the method described before with minor modifications [28].

### Anti-arthritis properties of EOS

#### Inhibition of albumin denaturation

In this assay, The percentage inhibition of protein denaturation was calculated following the reference [29, 30].

#### Freund’s Complete adjuvant (FCA)-induced arthritis in rats

Wistar rats (150–200 g) of either sex were used in this experiment. Grouping was the same as that illustrated above (Cotton pellet granuloma model). The experiment was carried out as described by [31, 32]. The rats were anesthetized and X-ray radiographs were recorded on a digital system.

#### Fluorescence Molecular Tomography (FMT)

A chronic in vivo arthritis model using FCA-induced mice was used to detect and quantify fluorescent agents in EOS-treated and non-treated groups of mice. The animals were injected subcutaneously with 0.03 ml of FCA into the plantar region of the left hind paw. Diclofenac sodium (5 mg/kg) was used as the positive control. EOS at 200 and 400 mg/kg were administered daily for 21 d. On day 21, 100 μL of ProSenes Perkin Elmer fluorescent imaging probe were injected into the tail vein of each mouse. After 24 h, the animals were anaesthetized using Phenobarbital. Each mouse was placed in the portable animal imaging cassette of the FMT and imaged using a Molecular Light Imager (Berthold Technologies) for 10 ms using a HQ 470 excitation filter (Chroma), HQ 525 emission filter (Chroma), and the WinLight32 software supplied with the instrument. Imaging was then performed using a two-step process and the WinLight32 software. First, a black and white photographic image was acquired using a 15 ms exposure. Next, the fluorescent image was acquired using a 5 min photon integration period with background subtraction. The fluorescent image was processed to colorize the fluorescence intensity, and then it was overlaid onto the black and white image for presentation. Quantification of the images was conducted, and the data were used to characterize the extent of the anti-arthritic effect and protection provided by EOS against the FCA-induced arthritis [33, 34].

### Statistical analysis

Data obtained from in vitro and in vivo experiments are expressed as mean ± SD. Statistical differences between the treatments and the control were evaluated by one-way analysis of variance followed by Tukey’s multiple comparison test using IBM SPSS software (Version 20). Differences were considered to be significant at *p* < 0.05 and *p* < 0.01.

## Results

### EOS contains high amounts of total phenolic and total flavonoid compounds

The total phenolic content of EOS (expressed as mg gallic acid equivalent per gram of EOS) was 385.092 ± 1.4 μg/mL. The total flavonoid content (mg quercetin equivalent per gram of EOS) was 57.185 ± 3.0 μg/mL. The result is presented as mean ± SD of 3 independent experiments in 3 replicates each.

### Free radical scavenging capabilities of EOS

The total reducing capacity of EOS was measured using the FRAP assay, which is based on a compound’s ability to reduce the Fe^3+^/tripyridyltriazine complex to a ferrous form (blue colored). The results of the FRAP study showed that EOS at 1.0 ± 0.1 μg/ml had effective reducing power. Similarly, scavenging ability of EOS was evaluated using the DPPH and ABTS assays. EOS had potent DPPH quenching activity (IC_50_ 14.87 ± 1.2 μg/ml) and ABTS quenching activity (IC_50_ = 15.98 ± 1.3 μg/ml). The standard reference, vitamin C, had IC_50_ values of 9.8 ± 0.38 and 4.2 ± 0.4 μg/ml in the DPPH and ABTS assays respectively (Table 1). The result is presented as mean ± SD of 3 independent experiments in 3 replicates each.

**Table 1:** Results of the DPPH, ABTS, and FRAP assays

Test	DPPH	ABTS	FRAP
EOS (μg/ml)	14.87 ± 1.2	15.98 ± 1.3	1.0 ± 0.1
Vitamin C (μg/ml)	9.8 ± 0.38	4.2 ± 0.4	-

### Anti-inflammatory effects of EOS

#### Effect of EOS on proliferation of U937 cells

The antiproliferative potency of EOS was evaluated using the U937 cell line and the MTT assay. EOS significantly inhibited proliferation of U937 cells (IC_50_ 91.0 ± 2.3 μg/ml). The result is presented as mean ± SD of 3 independent experiments in 3 replicates each.

#### In vitro inhibitory effect of EOS on production of IL-1 and TNF-α in U937 cells

ELISA was used to assess the in vitro effect of EOS on the inflammatory mediators in LPS-stimulated macrophages (i.e., U937 cells). EOS caused moderate significant (*p* < 0.05) inhibition of the levels of the proinflammatory cytokines IL-1 and TNF-α (IC_50_ 88 ± 1.9 and 63 ± 1.4 μl/ml, respectively). EOS also showed a dose-dependent inhibitory effect on IL-1 and TNF-α levels in these cells. The data is presented as mean ± SD of 2 independent experiments in 5 replicates each. *** *p* < 0.001, ** *p* < 0.01, * *p* < 0.05 vs. negative control.

#### In vitro inhibitory effect of EOS on COX-1 and COX-2 activities

**Fig. 1:**
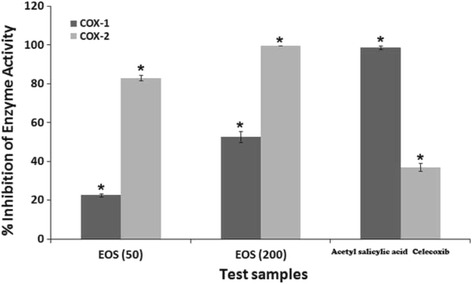
Results of the prostaglandin concentration in vitro colorimetric assay of the effects of EOS and standard drugs on the activities of COX-1 and COX-2 enzymes. Two EOS concentrations were used (50 and 200 μg/mL). The standard drugs used were acetyl salicylic acid (0.1 μM) for COX-1 whereas, celecoxib (0.05 μM) for COX-2. The results are presented as mean ± SD of 2 independent experiments in 5 replicates each. *** *p* < 0.001, ** *p* < 0.01, * *p* < 0.05 vs. control

The anti-inflammatory effect of EOS was determined using in vitro COX inhibition assay kits. EOS strongly inhibited both COX-1 and COX-2 activities. The inhibition of COX-1 by EOS at 50 and 200 μg/mL was 22.7 ± 0.8% and 52.6 ± 2.8%, respectively, whereas the values for COX-2 were 83.1 ± 1.3 and 99.7 ± 0.02%, respectively. The standard reference, celecoxib (0.05 μM) displayed potent inhibitory effect on COX-2 with 37 ± 2% whereas, acetyl salicylic acid (0.1 μM) showed 98.8 ± 1% on COX-1 (Fig. 1).

#### Inhibitory effect of EOS on carrageenan-induced paw edema in rats

The carrageenan-induced rat hind paw edema model was used to assess the anti-inflammatory effect of EOS in comparison with the standard reference drug indomethacin. The results show the percent inflammation recorded 3 h after carrageenan administration in the rats from different test groups. Inflammation was drastically reduced in the treated groups. The percent inflammation values of treatment with 100, 200, and 400 μg/ml of EOS were 49 ± 2.2%, 67 ± 1.8%, and 78 ± 2.7%, respectively, and indomethacin treatment resulted in 58 ± 3% inhibitions at 5 mg/kg. The data is presented as mean ± SD of 2 independent experiments in 6 replicates each. ** *p* < 0.01, * *p* < 0.05 vs. negative control.

#### Inhibitory effect of EOS on cotton pellet-induced granuloma in rats

**Fig. 2:**
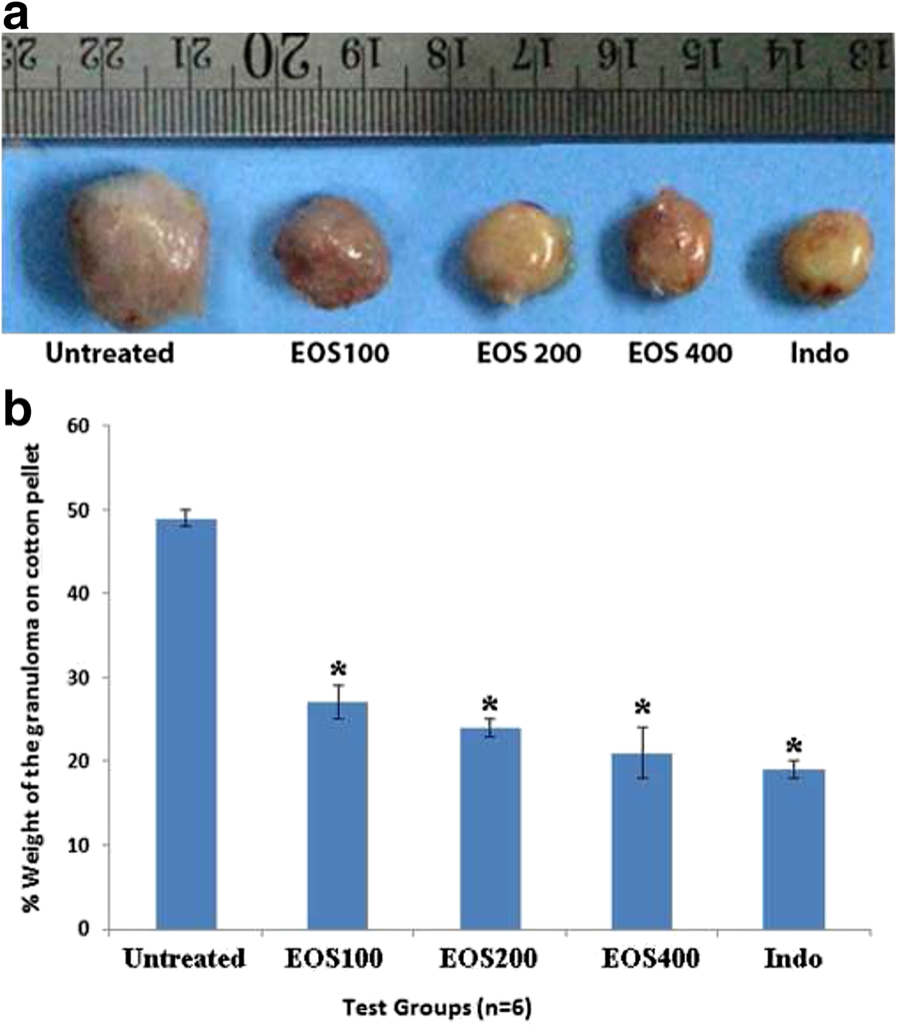
**a** Size of cotton pellets differed between control and treated animals. **b** Average Granuloma weight in the respective test groups also differed. Indomethacin was used as the positive control. The data is presented as mean ± SD of 2 independent experiments in 6 replicates each. * *p* < 0.05 vs. negative control

Granulation tissue was induced by subcutaneous implantation of cotton pellets in rats. Treatment with EOS significantly inhibited the inflammation in rats. The percent inhibition values of granuloma tissue were 15.8, 21.6, and 34.5% for EOS doses of 100, 200, and 400 μg/kg, respectively. Indomethacin and dexamethasone significantly inhibited granuloma tissue by 38.6 and 57.2%, respectively. Treatment with different amounts of EOS produced statistically significant dose-dependent inhibition of weight of the cotton pellet (*p* < 0.001). Figure 2 shows mean weight of the cotton pellets dissected from the tested rats.

#### In vivo inhibitory effect of EOS on production of IL-1 and TNF-α in rats

**Fig. 3:**
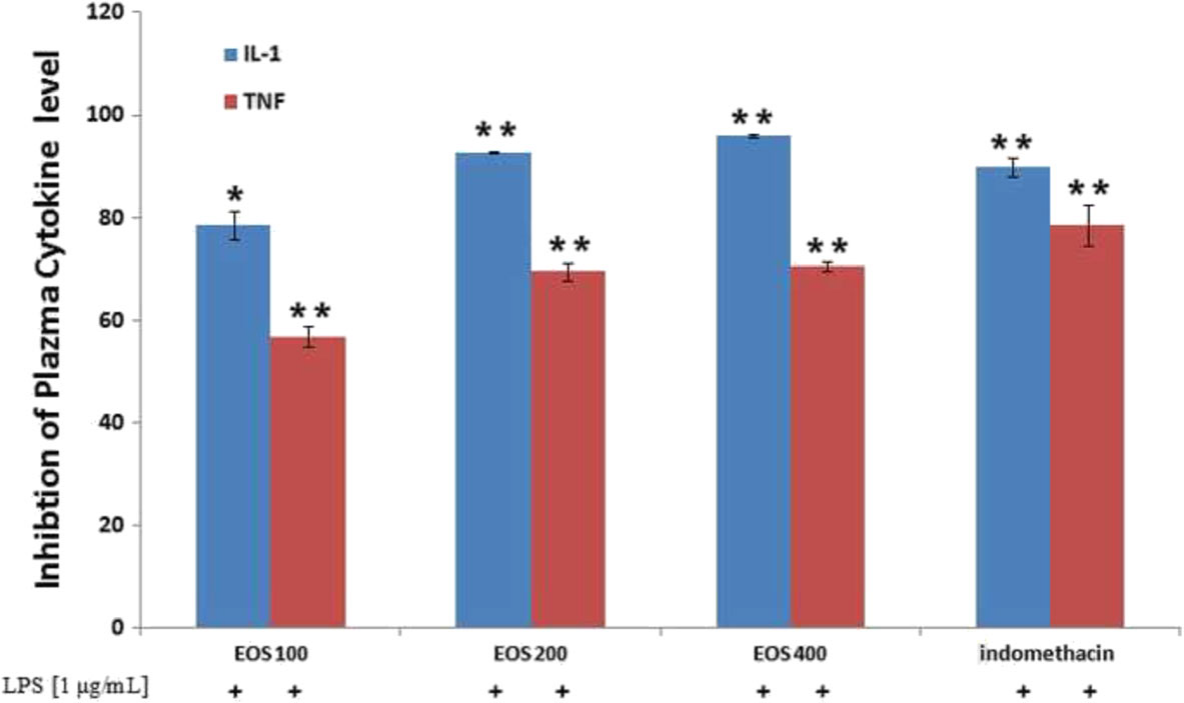
Inhibitory effect of EOS (100, 200, and 400 mg/kg) and the standard drug (indomethacin) on the activities of TNF-α and IL-1 in the plasma samples of rats. The results are presented as mean ± SD of 2 independent experiments in 5 replicates each. * *p* < 0.005, ** *p* < 0.01 vs. control

Levels of the inflammatory mediators IL-1 and TNF-α in blood collected from the treated and control animals were measured using ELISA. The results were in agreement with that of the in vitro study described above. Treatment with EOS caused a significant decrease in the contents of these serum inflammatory mediators when compared to the control (*p* < 0.01). EOS treatment also showed dose-dependent inhibitory effects. At 100, 200, and 400 mg/kg of EOS, the levels of IL-1 were 78.6 ± 2.7, 92.7 ± 0.1, and 96 ± 0.2%, respectively, compared with indomethacin (89.8 ± 1.9%). The percent inhibition of serum TNF-α by the 100, 200, and 400 mg/kg doses of EOS were 56.8 ± 2, 69.5 ± 1.6, and 70.5 ± 1%, respectively. The levels in the treatment groups were comparable to that of the standard reference, indomethacin (78.5 ± 4) (Fig. 3).

### Anti-arthritis properties of EOS

#### Inhibitory effect of EOS on albumin denaturation in vitro

Anti-arthritis activity was studied using an in vitro inhibition of protein denaturation assay, as the relationship between protein denaturation and arthritis was documented recently. In vivo denaturation of protein leads to increasing production of auto-antigen, which causes the disease. The three concentrations of EOS (100, 200, and 400 μg/ml) tested provided significant protection against protein denaturation (78.8 ± 1, 88.5 ± 3.6, and 93.7 ± 3.4%, respectively). Acetyl salicylic acid (250 μg/ml) resulted in 42.7 ± 3.1% inhibition of protein denaturation (Table 2).

**Table 2:** Percentage inhibition of protein denaturation by three concentrations of EOS

Groups (μg/ml)	Protein denaturation (%)
EOS 100	78.8 ± 1*
EOS 200	88.5 ± 3.6***
EOS 400	93.7 ± 3.4**
Acetyl salicylic acid	42.7 ± 3.1

Acetyl salicylic acid (250 μg/ml) was used as the positive control. The data is presented as mean ± SD of 3 independent experiments in 3 replicates each. *** *p* < 0.001, **p < 0.01, *p < 0.05 vs. negative control

#### Inhibitory effect of EOS on FCA-induced paw edema in rats

**Fig. 4:**
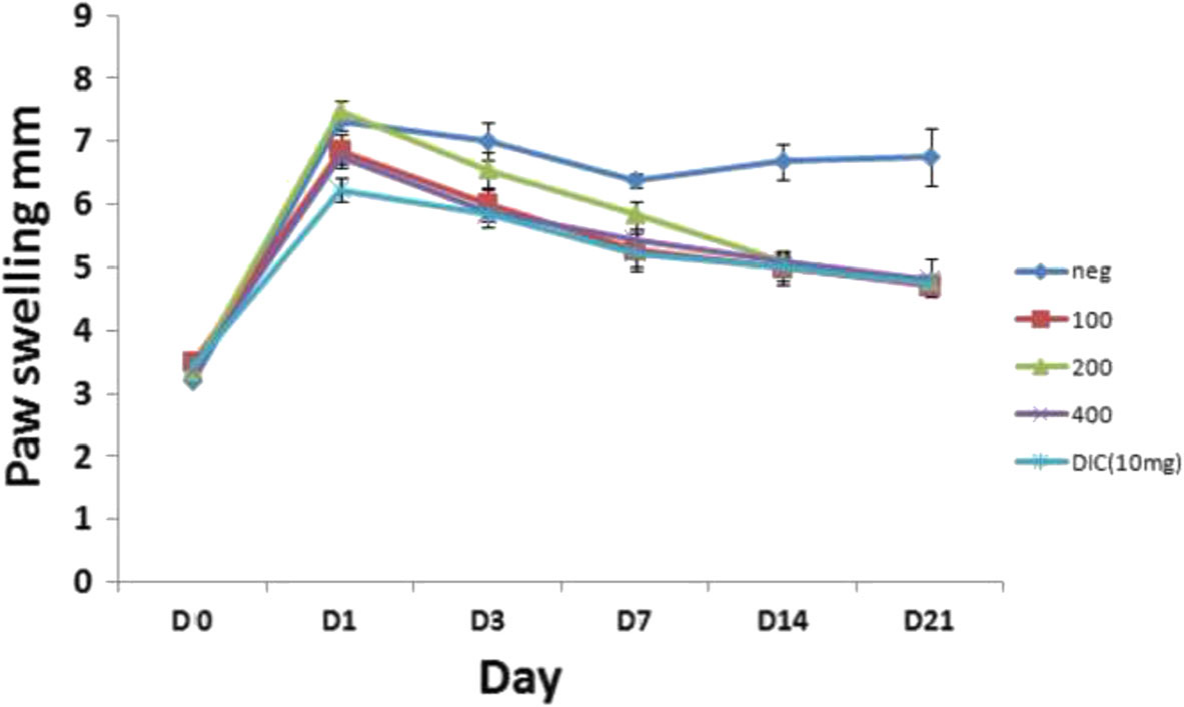
The effect of EOS on paw swelling (mm) in rats induced by FCA during 21 d of treatment. Treatment with EOS resulted in profound inhibition of the tibio-tarsal joint diameter compared with negative control animals. The results are presented as mean ± SD of 2 independent experiments in 6 replicates each

Injection with FCA of the right hind paw of rats leads to an increase in the size of the joint. Maximum joint swelling was observed in all groups on the second day after immunization. Chronic treatment with EOS effectively reduced the lesions in arthritic rats. The major reduction of joint swelling was detected on day 21 in rats treated with EOS and the positive control (Fig. 4).

#### Radiographs of the paw 21 d after treatment with FCA

**Fig. 5:**
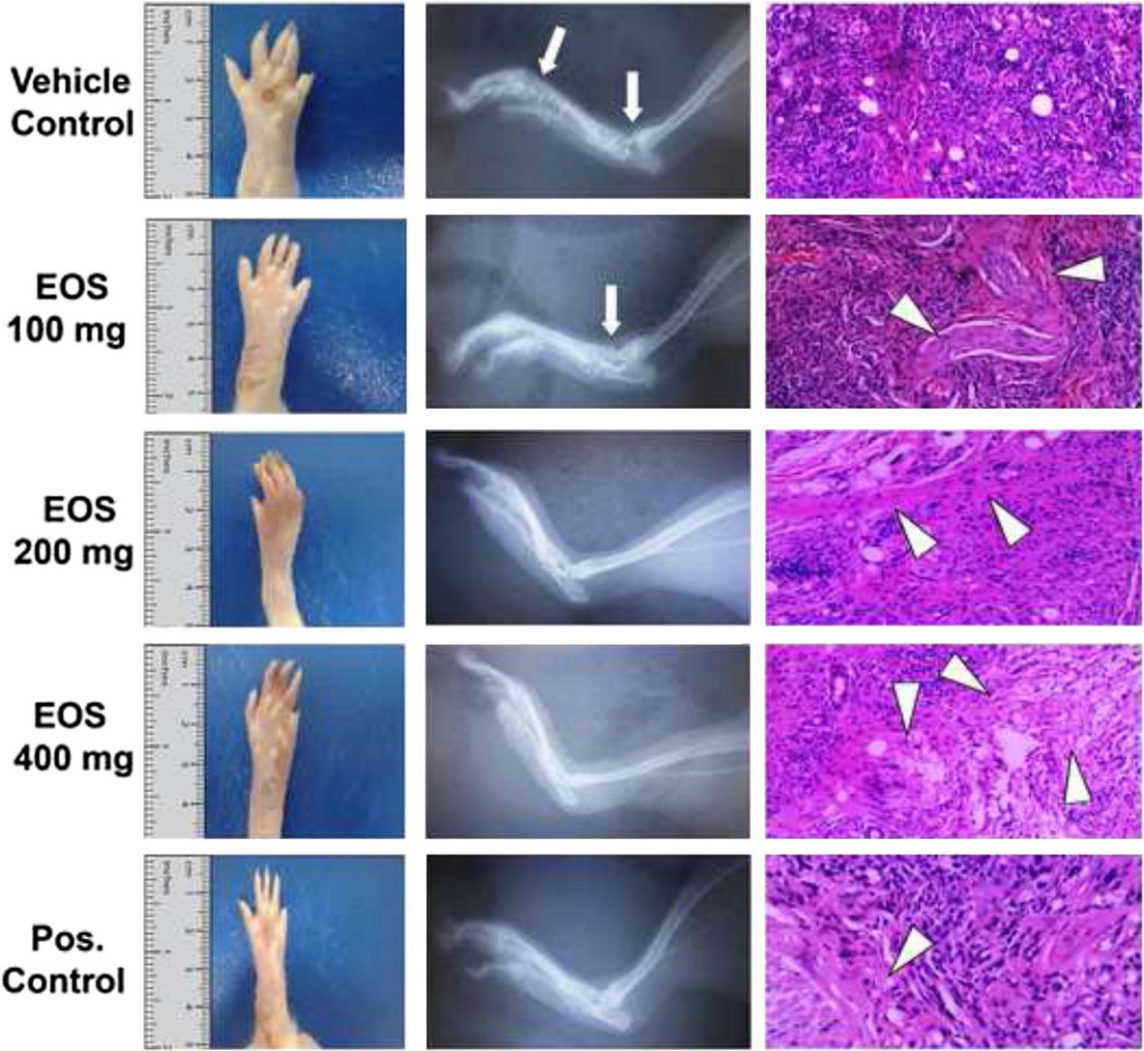
Anti-arthritic efficacy of EOS in FCA-induced arthritic rats. **a** Clinical symptoms (*left panels*), **b** X-rays (*middle panels*), and **c** histological analysis (*right panels*, hematoxylin and eosin stained) of FCA-induced arthritic rats treated with distilled water (negative control), different doses of EOS (100, 200, and 400 mg/kg), and diclofenac sodium (positive control). FCA injection induced severe inflammation and bone destruction, which were evidenced via paw edema, bone degeneration, and osteoporotic lesions (*arrows*). Treatment with EOS suppressed hind paw swelling and bone destruction. Histological sections from the negative control group showed severe synovitis marked by the loss of cartilage tissue and the presence of numerous macrophages. Total score for synovitis recorded for the negative control group is 13 points. However, treatment with EOS significantly reduced the number of macrophages and increased the production of collagen fibrils (arrow heads). Total score for synovitis recorded for 100, 200 and 400 mg/kg doses of EOS are 10, 7 and 3 points, respectively. The anti-arthritic effect of EOS was comparable to that of diclofenac sodium (total score for synovitis recorded is 4 points). The data is presented as mean ± SD of 2 independent experiments in 6 replicates each

X-rays of the synovial joint of the hind leg of FCA-treated rats revealed significant numbers of osteoporetic lesions and bone degeneration. The X-ray image shows marginal swelling in the bone and slight destruction of the cartilage 21 d after treatment (Fig. 5b). In contrast, the radiographic images of rats treated with high doses (200 and 400 mg/kg) of EOS and those treated with diclofenac sodium showed no callus formation, deformity, or irregular margins of cartilage and bone, which illustrates the significant pharmacological anti-inflammatory action of EOS compared to the control (Fig. 5b).

#### Histopathological changes of the paw 21 d after treatment with FCA

The rats were sacrificed via anesthesia on day 21. Knee joints and hind paws were removed from the rats and fixed in 10% PBS, Serial paraffin sections (5 mm) were stained with hematoxylin and eosin. The rats treated with EOS and diclofenac sodium showed significant reduction in synovial hyperplasia, inflammation, and erosion of the synovial membrane and cartilage compared with the control group (Fig. 5c).

The degree of histologic changes was graded semiquantitatively on a scale of 0-3 points [35], 0 corresponding to “not present, i.e. normal”, whereas 1 = mild, 2 = moderate, 3 = severe. Accordingly, the changes in each test group were then summed up. The pathological characteristics of synovial membrane included were as follows,

1. Hyperplasia in Synovial membrane.
2. Presence of multinucleated Synovial giant cells.
3. Presence of inflammatory cell.
4. Presence of granuloma tissue including destruction of the articular cartilage and bone.
5. Loss of collagen fibrils or presence of necrosis.

#### FMT

**Fig. 6:**
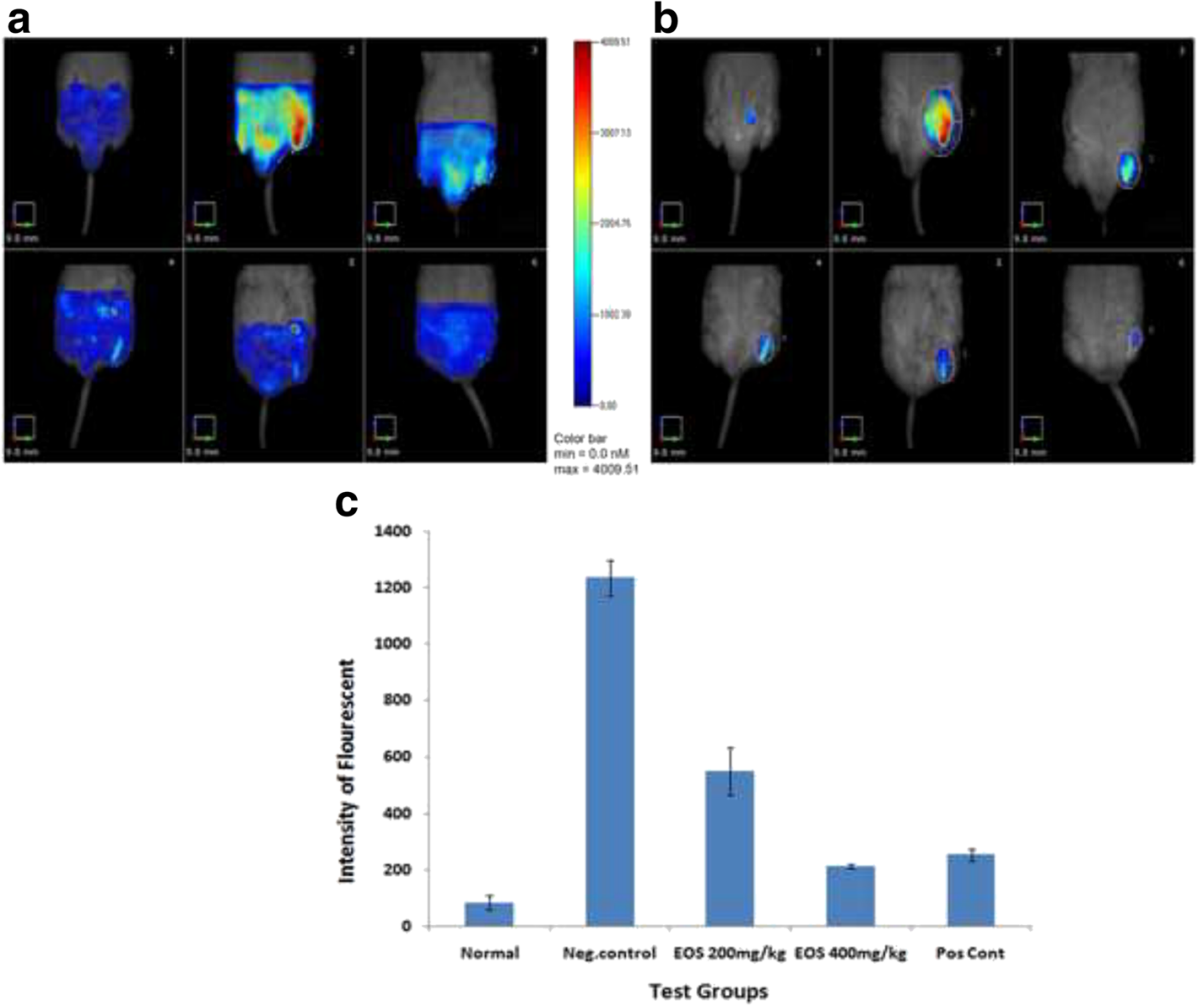
**a** & **b** FMT tomographic imaging or rat paws to assess arthritis disease severity using ProSense fluorescent imaging agent. (1 & 6) Tomographic images of normal control without FCA treatment. (2) The fluorescent signal of the negative control is represented as a maximum intensity projection. (3) Result for the group treated with the low concentration (200 mg/kg) of EOS. (4) Results for the group treated with the high concentration (400 mg/kg) of EOS. (5) Result for the group treated with the positive control, diclofenac sodium (5 mg/kg). **c** Quantifications of paw fluorescence for each of the two EOS doses and the positive and negative controls. The data is presented of 2 replicates each

FCA elicits inflammation by stimulating proinflammatory mediators, thus it can provide quantitative information about inflammatory joint disease development in the mouse RA model. It is well know that cathepsins are upregulated in inflammatory diseases particularly in rheumatoid arthritis. In the present study, FMT system was used to quantify the cathepsins which are the specific aspects of rheumatoid arthritis and osteoporosis. The FMT scanning analysis showed significantly high intensity of fluorescent signal in the negative control animals bearing chronic inflammation induced by the FCA. Whereas, treatment with EOS showed a dose-dependent reduction of the fluorescence intensity. These results can be compared with the standard reference drug, indomethacene (Fig. 6).

## Discussion

*O. stamineus* is a traditional medicinal herb with various and dynamic medicinal properties [9, 36]. It has been used in traditional medicines to treat inflammatory-related disorders such as renal nephritis, cancer, and arthritis [8]. Although the plant is widely used as a folk remedy to treat arthritis and several in vitro studies of the anti-inflammatory properties of the herb have been reported [37]. Two compounds have anti-inflammatory properties, pimarane diterpenes, orthosiphol A and B, have been isolated from the plant [12], mechanistic studies of its effects on arthritis using a suitable in vitro and in vivo model are lacking. Thus, the present study was conducted to investigate the anti-arthritis properties of EOS and to correlate the in vitro antioxidant and anti-inflammatory potentials of EOS with its inhibitory effect on paw edema and granuloma in rats.

### Antioxidant activities of EOS

Antioxidants are compounds that protect cells against the damaging effects of reactive oxygen species such as peroxynitrite, superoxide, singlet oxygen, hydroxyl, and peroxyl radicals. Cellular damage occurs due to the imbalance between antioxidants and reactive oxygen species, which takes place because of exposure of a biological system to highly reactive free radicals generated by exogenous agents (e.g., radiation, chemicals, hyperoxia) and endogenous processes such as normal cellular metabolism [38]. It is linked to inflammation, cancer, atherosclerosis, aging, ischemic injury, and neurodegenerative diseases [39]. Natural antioxidants have garnered increasing interest among consumers and the scientific community because epidemiological studies have indicated a lower risk of cardiovascular disease and cancer when natural antioxidants are consumed frequently [40, 41]. We found that EOS has potent antioxidant activities based on its capacity to reduce ABTS^+^ (IC_50_ 16.16 μg/ml), its ability to reduce the Fe^3+^/tripyridyltriazine complex to its blue-colored ferrous form (FRAP) (1.0 μg/ml), and its ability to scavenge DPPH (IC_50_ 14.8 μg/ml) (Table 1).

The special effects of phenolic and flavonoid compounds in plants on antioxidants also have been reported. The redox properties of these compounds are responsible for their main effects. EOS contains high amounts of total phenolic and flavonoid compounds (350 ± 0.6, 131 ± 0.9, respectively).

### Anti-inflammatory activity of EOS

The present study was conducted to investigate the anti-inflammatory potential of EOS, which likely is responsible for the wide range of pharmacological properties of this herb, and to correlate its in vitro antioxidant and anti-inflammatory properties with its inhibitory effect on paw edema and granulation tissue in rats. Proinflammatory mediators such as TNF-α and IL-1 play significant roles in the pathophysiology of arthritis [42]. TNF-α is one of the most important inflammatory cytokines, as it controls different types of cell functions. The overproduction of TNF-α is linked to the development of various diseases such as asthma, RA, psoriatic arthritis, inflammatory bowel disease, septic shock, diabetes, and atherosclerosis [43]. Inflammatory mediators such as IL-1 are also essential in synovcyte destruction in different immunoinflammatory diseases such as RA [44]. Both TNF-α and IL-1 induce the production of high levels of matrix metalloproteinases by synovial cells and chondrocytes [45]. COX-2 is highly inducible by serum, growth factors, lipopolysaccharides, and cytokines, especially IL-1, in certain cell types involved in the inflammatory processes (e.g., fibroblasts and macrophages). IL-1β increases the synthesis of COX-2, which plays a significant role in the overproduction of prostaglandin E_2_, thereby leading to arthritis [46].

In the present study, treatment with EOS resulted in a significant in vitro reduction of IL-1 and TNF-α levels (IC_50_ values of 88 ± 1.9 and 63 ± 1.4 μl/ml, respectively) as well as in vivo dose-dependent inhibitory effects (Fig. 3). On the other hand, TNF-α and IL-1 collectively interact with COX-2 to activate the immune system. Induced inflammation in the in vivo animal model suggests that the produced edema is associated with the accumulation of COX-2 mRNA and thromboxane. As a result, inhibition of COX-2 is considered to be a main approach to treating inflammation [47]. In the current study, EOS treatment caused significant suppression of COX-1 and mainly COX-2 activities (52.6 ± 2.8% and 99.7 ± 0.02%, respectively). Moreover, treating the carrageenan-induced edema in the rat paw with EOS resulted in a potent reduction of swelling and inflamed paw. These results suggest a correlation between the anti-inflammatory ability of EOS and its effectiveness in reducing COX-2 levels. The inhibitory effects of COX-2 give EOS a high therapeutic safety used to avoid gastrointestinal (GI) adverse effects of other ant-inflammatory drugs [48]. To evaluate the efficacy of the extract against the later proliferative phase of inflammation that results in tissue degeneration and proliferation of macrophages, neutrophils, fibrosis, and multiplication of small blood vessels, we conducted the cotton pellet granuloma test. The granuloma test is a dependable in vivo model that is widely used to study chronic inflammation [49]. EOS treatment resulted in decreased granuloma weight, which indicates that the extract suppressed cell proliferation (Fig. 2).

### Anti-arthritis activity of EOS

Arthritis is a chronic inflammatory disease that targets all joints in the body causing erosions in the lining of the joint [50]. Several models to study arthritis have been reported. One of the most used is called FCA-induced arthritis. It assesses subchronic or chronic inflammation in rats and is of considerable relevance for the study of pathophysiology and pharmacological control of inflammatory processes [51]. This model acts by lengthening the life span of the injected autoantigen, stimulating autoantigen delivery to the immune system, and giving a group of signals to the innate compartment of the immune system, resulting in proliferation and differentiation of leukocytes [52]. The efficacy of anti-arthritic drugs and herbal products can be tested and validated using parameters such us paw swelling measurement, cytokine profiles, chemokine profiles, bone remodeling molecules, functional disability, spleen weight, X-ray, and histopathology of the paw [53, 54].

Several medicinal plants have been reported for their potential application in the treatment of arthritic inflammation and bone damage, and herbal remedies are widely used in preclinical and clinical studies [31, 55]. In the current study, EOS demonstrated potent in vitro and in vivo anti-arthritic activity. It protected against denaturation of protein, which controls the production of autoantigen and is considered to be one of the basic causes of RA (Table 2). Denaturation may involve alteration of electrostatic, hydrogen, hydrophobic, and disulphide bonding [56]. The effect of EOS could be due to the presence of phenolic and flavonoid compounds and to the high antioxidant activities of EOS (Table 1).

In vivo findings showed that EOS suppressed the chronic phase of inflammation when compared with the FCA control group. The animals treated with EOS (100, 200, and 400 mg/kg) and diclofenac sodium (5 mg/kg) showed a significant reduction in the size of the Granuloma

After 21 d of treatment, the inflammatory mediators COX-1/2 and IL-1β in the synovial membrane are closely associated with the degree of edema. [57]. The changes in the left paw edema size in EOS treatment vs. control groups over time showed that EOS strongly inhibited inflammation, likely due to the presence of high amounts of flavonoid and phenolic compounds. In addition, EOS treatment strongly suppressed inflammatory mediators such as IL-1, TNF-α, COX-1 and COX-2 as described above. The radiographic images of the negative control animals showed swelling, destruction of cartilage, and erosion of the synovial membrane and articular meniscus. However, groups treated with the positive control and EOS (200 and 400 mg/kg) did not show any of these signs. Moreover, histopathological evaluation of the treated groups showed no synovial hyperplasia or erosion of the synovial membrane and cartilage relative to the control group.

FMT has been used to assess tumor, inflammatory, pulmonary, cardiovascular, and skeletal diseases. This technique provides non-invasive, whole body, deep tissue imaging in small animal models, and in this study it generated three-dimensional information-rich results to evaluate the efficacy of EOS against chronic inflammation and arthritis [58]. The pros of using FMT are that it provides a good understanding of the mechanism and progression of diseases and it supports results from other models. It yields sensitive readouts regarding the pathology of the disease, and that it may prove possible to apply FMT results to predict disease development. It has been already proved that Cathepsin plays a crucial role in pathogenesis of different disorders of inflammation specifically rheumatoid arthritis (RA) whereas it may cause bone erosion and cartilage degradation in RA joints, increasing the activity of Cathepsin in synovial membranes involves potentially in cartilage degradation and bone destruction [59]. Cathepsin is mainly induced by inflammatory mediators such as TNF-α, IL-1 and –6. In the current study, we have proven the significant reduction of TNF-α, IL-1. As ProSenes is activated by the key disease associated proteases, Cathepsin, FMT system was applied to quantify the cathepsins to study disease progression and therapeutic response in animal models of arthritis. FMT results showed significantly reduction in the intensity of fluorescent signal in the treated groups which is resembles the reduction of cathepsins. Our findings were consistent with the histology and X-ray results.

## Conclusion

In conclusion, treatment with EOS significantly suppressed acute and chronic inflammation induced in rats. Treatment of FCA-induced arthritic rats with EOS also provided protection of bone, cartilage, and soft tissues from inflammatory damage. Histological analysis revealed regeneration of collagen fibrils and suppression of macrophage levels at the inflammatory site in EOS-treated animals. In addition, EOS treatment reduced the levels of inflammatory markers such as TNF-α, IL-1, COX-1, and COX-2 in human macrophages and arthritic rats, suggesting that EOS may suppress these inflammatory mediators. Thus, results of this study demonstrate a potential use of *O. stamineus* for the treatment of arthritis and other inflammatory disorders.

## Abbreviations

COX: Cyclooxygenase
EOS: 50% ethanol extract of *O. stamineus* leaves
FCA: Freund’s Complete adjuvant
FMT: Fluorescence molecular tomography
IL-1: Interleukin 1
NSAIDs: Non-steroidal anti-inflammatory drugs
RA: Rheumatoid arthritis
TNF-α: Tumor necrosis factor
TPA: (12-O-tetradecanoylphorbol-13-acetate)
